# Supplementary material for: Establishing multiple omics baselines for three Southeast Asian populations in the Singapore Integrative Omics Study
Source: Nat Commun. 2017 Sep 21;8:653. doi: 10.1038/s41467-017-00413-x (PMC5608948; doi:10.1038/s41467-017-00413-x)
Supplement: Supplementary file 1 — Supplementary Information [file 41467_2017_413_MOESM1_ESM.pdf]

File name: Supplementary Information
Description: Supplementary figures, supplementary tables, supplementary methods and supplementary references.

File name: Supplementary Data 1
Description: List of MRM transitions measured in this study

File name: Supplementary Data 2
Description: List of 520 candidate genomic regions that are significantly differentiated between the three ethnic groups

File name: Supplementary Data 3
Description: List of 280 significantly differentiated transcript probesets between the three ethnic groups, after adjusting for gender and laboratory batch effects

File name: Supplementary Data 4
Description: List of 107 significantly differentiated lipids between the three ethnic groups, after adjusting for gender

File name: Peer review file
Description:

Supplementary Methods

1. Genomics

1.1 Combine Illumina Omni 2.5 SNPs and Illumina Exome Chip

A preliminary round of QC was performed on the SNPs as to identify a set of combined SNPs from two genotyping platform. This was performed independently for the both datasets. Five criteria in the stated order were used to identify SNPs for exclusion when combining SNPs from two microarray: (i) unknown or duplicate genomic coordinates (12,919 SNPs in Omni 2.5 and 879 SNPs in Exome Chip); (ii) unknown strand information (3 SNPs in Omni 2.5 and no SNP in Exome Chip); (iii) not in autosomal and X/Y chromosomes (256 SNPs in Omni 2.5 and 226 SNPs in Exome Chip); (iv) conflict in allele designation between the two SNPs dataset (60 SNPs in Omni 2.5 and 60 SNPs in Exome Chip); (v) genotype concordance <99.5% for both datasets (160 SNPs in Omni 2.5 and 160 SNPs in Exome Chip). This removed a total of 13,398 SNPs out of the total of 2,379,855 SNPs in Omni 2.5 and a total of 1,325 SNPs out of the total of 274,959 SNPs in Exome Chip. The total of combined SNPs is 2,600,320 SNPs.

1.2 Sample QC

The quality of the genotype data for each sample was assessed using the SNPs that combined after the combine round of SNP QC. This was performed independently for each population. Samples were identify for removal in the following criterias: (i) high missingness (>2%); (ii) excessive identify-by-state (IBS) genotypes or genenetically inferred to be outliers with principal component analysis (PCA) from the self-reported population membership. A total of 1 sample was removed out of 111 Chinese samples, 12 samples were removed out of 120 Malay samples and 14 samples were removed out of 119 Indian samples. The remaining population composition is 110 Chinese, 108 Malays and 105 Indians

1.3 SNP QC

Final round of SNP QC performed for each population on all the genetic data separately. The SNPs are removed on the basis of: (i) missingness >5%; (ii) HWE p-value <0.001; (iii) non-autosomal SNPs; (iv) indels. Hence, this resulted in a final set of 2,299,708 (Omni 2.5) and 227,750 (Exome chip) unique SNPs across 110 Chinese, 108 Malays and 105 Indians.

2. Lipidomics

2.1 Blood sample collection and plasma processing

For each plasma samples, total of 10ml of whole blood was collected by venipuncture in BD Vacuatiner® plastic plama tubes with K2 EDTA (#366643, lavender closure) that serves as an anticoagulant. After mixing of the blood with the anticoagulant, the blood was spun at 2,200g for 15 minutes in a pre-cooled, 4°C swing-out bucket rotor centrifuge (Allegra 6R Centrifuge, Beckman Coulter, Fullerton, CA). Next, the top plasma layer was removed by pipetting out the supernatant and placed in the cryovials (Practical Mediscience, Singapore) as to reduce the contamination by platelets. After that, all plasma samples were well mixed and frozen at -20°C. Subsequently, the plasma samples

were divided into 1 ml aliquots and stored at -80°C for extraction and lipid analysis via mass spectrometry. All the plasma samples underwent same number of freeze-thaw cycles as this is to ensure the consistency of sample processing.

2.2 Lipid extraction

For lipid extraction, the extraction process has been previously described[1]. The purchase of all the lipid internal standards (ISTDs) and solvents were followed as previously described[1,2] (**Supplementary Table 13**).

3. Transcriptomics

3.1 Probesets QC

A probesets QC was performed on the 33,297 transcript probesets (Affymetrix HuGene-1.0 array based on the NetAffx[3] na35 annotation) as to identify a set of unique and express in at least one sample for sample QC. Four criteria in the stated order were used to identify Affymetrix probes for exclusion: (i) no gene annotation probesets (based on gene_assignment); (ii) non-autosomal transcript probesets; (iii) transcript probesets with cross hybridization≥2 (based on cross_hyb=2 or 3); (iv) low expression probesets in all the samples (based on log2-intensity ≤6). This removed a total of 11,648 transcript probesets.

3.2 Samples QC

A total of 349 samples with 33,297 transcript probesets were evaluated during samples QC. Samples were identified for removal on the basis of: (i) RIN value ≤7.0 (61 samples were removed). RMA normalization[4] was then performed on a total of 288 samples as to get the normalized expression value for all samples. Subsequently, hierarchical clustering was conducted to identify outliers. For hierarchical clustering, the distance matrix is defined as “Euclidean” distance and the “complete” method is used for agglomerative clustering (**Supplementary Method 4.3**). (ii) Cut the tree at height = 90 with a criteria of minimum size of the branch is 10 (17 samples were removed). Sample differentiation was examined and was achieved through the use of clustering using the program “plotMDS” from the limma package[5] based on the top 500 transcript probes (**Supplementary Method 4.3**). We observed two distinct gender clusters from the MDS plot. (iii) samples with gender discordance between recorded genders and MDS plot (2 samples were removed).

There are 80 samples were removed out of possible 349 samples, and the population composition of the remaining 269 samples is: 98 Chinese, 75 Malays, 96 Indians.

3.3 Codes

<u>RMA normalization</u>

```
%	apt-probset-summarize –a rma –o Output_rma.txt –cel-files CEL_list
```

<u>Hierarchical Clustering</u>

```
%	library(WGCNA)
```

```
%	transposed_output_rma<- t(output_rma)
%	sampleTree <- hclust(dist(transposed_output_rma), method="complete")
%	clust = cutreeStatic(sampleTree, cutHeight=90, minSize=10)
%	keepSamples = (clust==1)
%	transposed_output_rma <- transposed_output_rma [keepSamples,]
```

<u>MDS Clustering</u>

```
%	library(limma)
%	expression_data<-t(transposed_out_rma)
%	gender.color<-data.frame(Gender=transcriptomics$gender,
color=labels2colors(transcriptomics$gender))
%	mds<- plotMDS(expression_data,gene.selection=”common”)
%	plot.MDS(mds,gender.color)
```

4. Statistical Analysis

<u>4.1 Principal component analysis for non-genomic data using R[9]</u>

For lipid data, gene expression data, miRNA data:

```
%	prcomp(data,retx=T,center=T,scale.=T)
```

For the combination of clinical, lifestyle and dietary data:

```
%	prcomp(data,retx=T,center=T,scale.=F)
```

For dietary data:

```
%	library(cluster)[10]
%	distance.matrix<-as.matrix(daisy(dietary_data,metric=”euclidean”,stand=F))
%	prcomp(distance.matrix,retx=T,center=T,scale.=T)
```

<u>4.2 Anova using R</u>

For lipid data:

```
%	p<-anova(lm(lipid ~gender + Ethnic_group))[[3]][2]
```

For gene expression data, adjusted for batch and gender:

```
%	p<-anova(lm(intensity~Batch + Gender + Ethnic_group))[[5]][3]
```

For continuous variable in the clinical, lifestyle and dietary data, adjusted for gender:

```
%	p<-anova(lm(intensity~ Gender + Ethnic_group))[[5]][2]
```

<u>4.3 Identifying tagging and tagged lipids using *CLUSTAG*[6]</u>

```
%	java -cp TaggingSetChooser.jar -mx500G
hk.hku.csis.biosphere.algorithm.TaggingSetChooser
sim=hk.hku.csis.biosphere.similarityscore.SimilarityMatrix link=C threshold=0.8
data=rawld.txt scale=1000 corr=<Chinese_LD.txt> pos=<Chinese_info.txt>
map=results/TaggingSetChooser.result.MaxMinLink.html
```

mem=results/TaggingSetChooser.result.MaxMinLink.members result=<result.txt> > logfile

Supplementary Figures

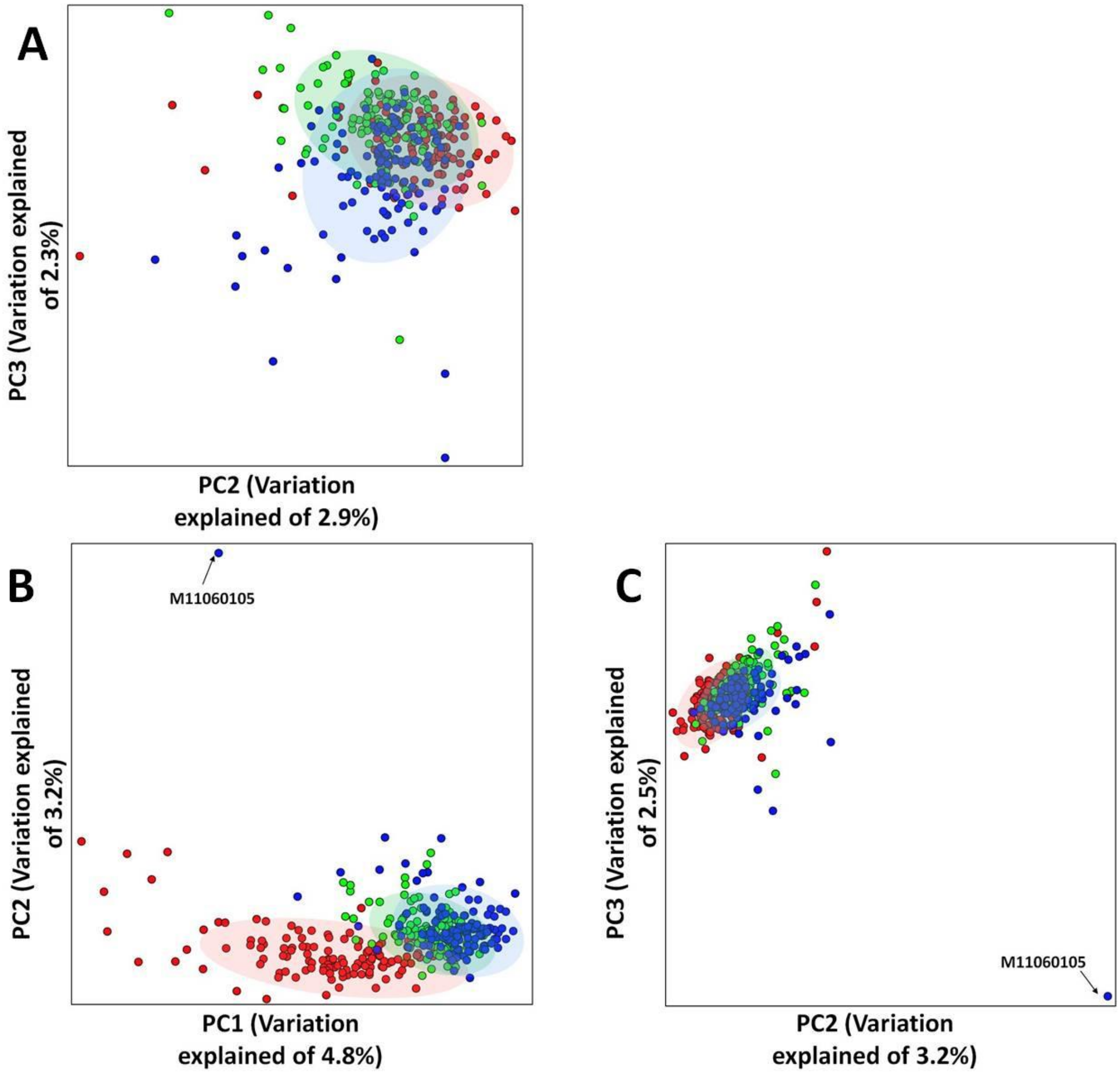

Supplementary Figure 1. Sample-level principal component analysis on the 199-food items dietary data

Biplots are shown for **(A)** the second and third axes of variations from eigen-decompositions across 199 dietary variables across 122 Chinese, 116 Malays, and 119 Indians; (**B & C**) the first three axes of variations from eigen-decompositions across 199 dietary variables across 122 Chinese, 116 Malays and 120 Indians. We observed an outlier (M11060105) from the biplots and it was removed for the PCA in **Figure 1F** and **Supplementary Figure 1A**. Each circle represents an individual from a particular ethnic group and is assigned a colour unique to that ethnic group that is represented in legend on bottom right panel in **Figure 1.**

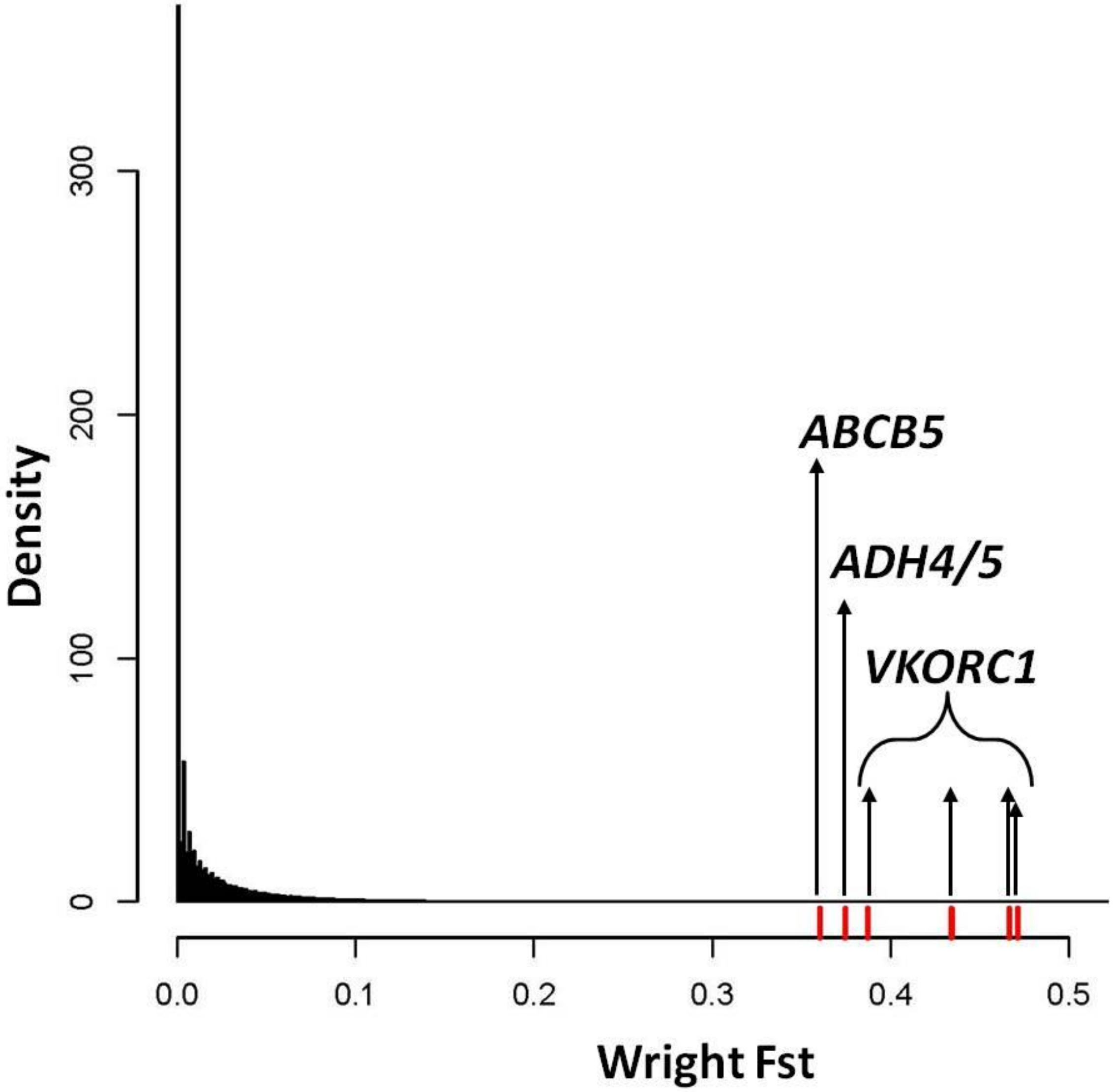

Supplementary Figure 2. 6 pharmacogenomic variants distribution that encoded for *VKORC1* gene in three ethnic groups

The distribution of the 6 pharmacogenomic variants that encoded for *VKORC1, ADH4/5 and ABCB5* genes. The Wright's Fst value and the frequency of the variant in each ethnic group can be found in **Table 2**.

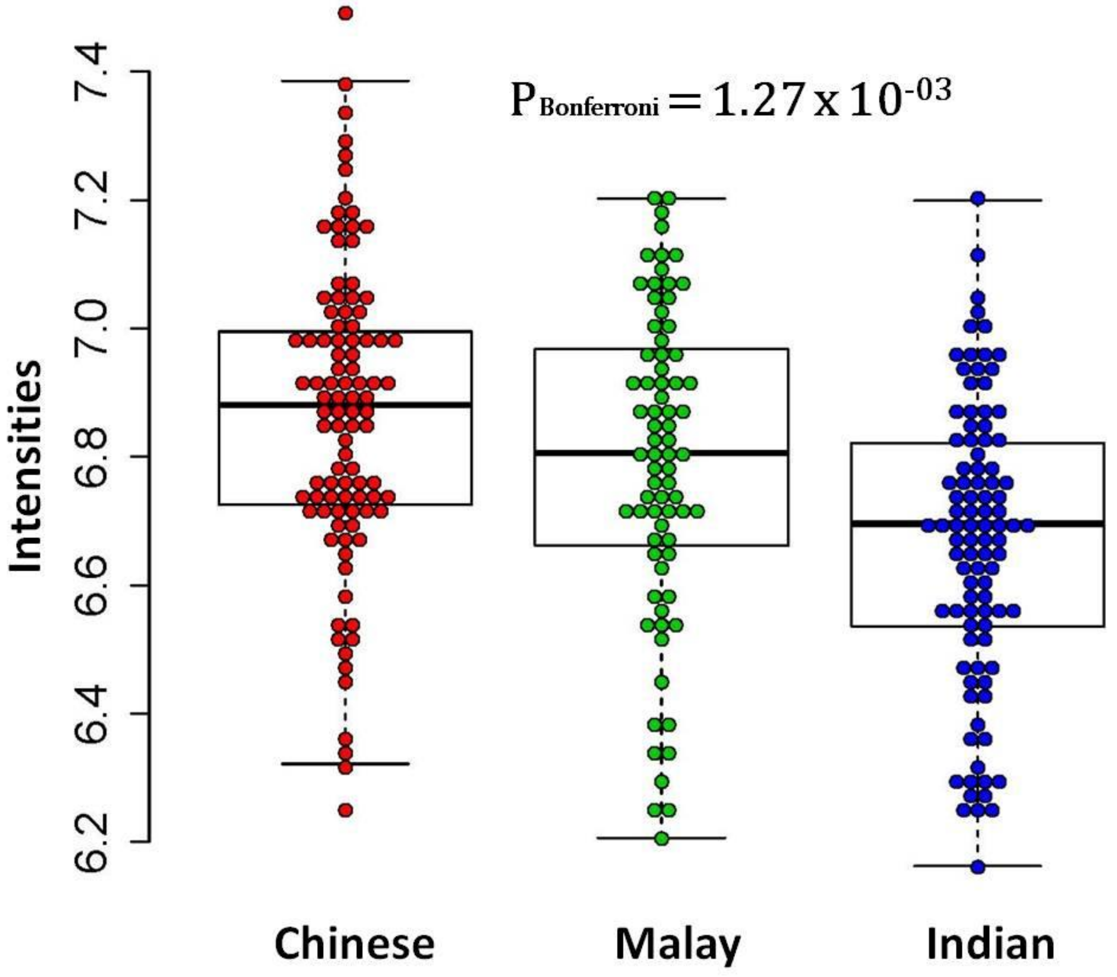

Supplementary Figure 3. The scatter plot of 8015769 transcript, annotated for *BRCA1* gene in 3 ethnic groups

The scatterplot of 8015769 probesets that encoded for *BRCA1* gene that related to breast cancer in 3 ethnic groups.

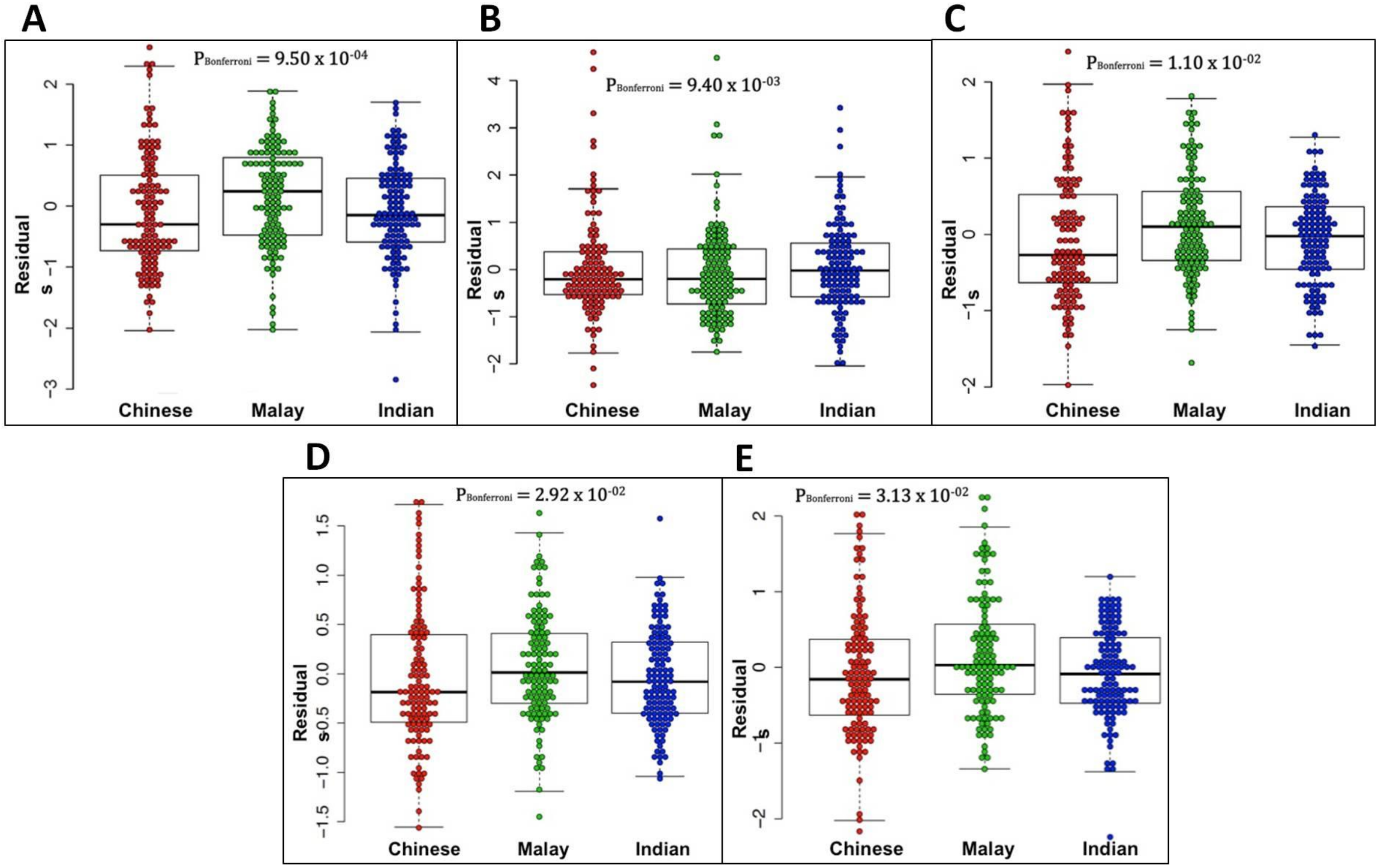

Supplementary Figure 4. A combined boxplot and scatter plot of the five significant miRNAs across 3 ethnic groups. The combined plot of the distribution of residuals of miRNAs copy number of A) *hsa-miR-4732-3p*; B) *hsa-miR-375*; C) *hsa-miR-140-3p*; D) *hsa-miR-378a-3p*; and E) *hsa-miR-378a-5p* across three ethnic groups. *P*-values were calculated using ANOVA, adjusted for batch effect and corrected for Bonferroni. The details of the significant miRNAs across 3 ethnic groups can be found in **Table 4**.

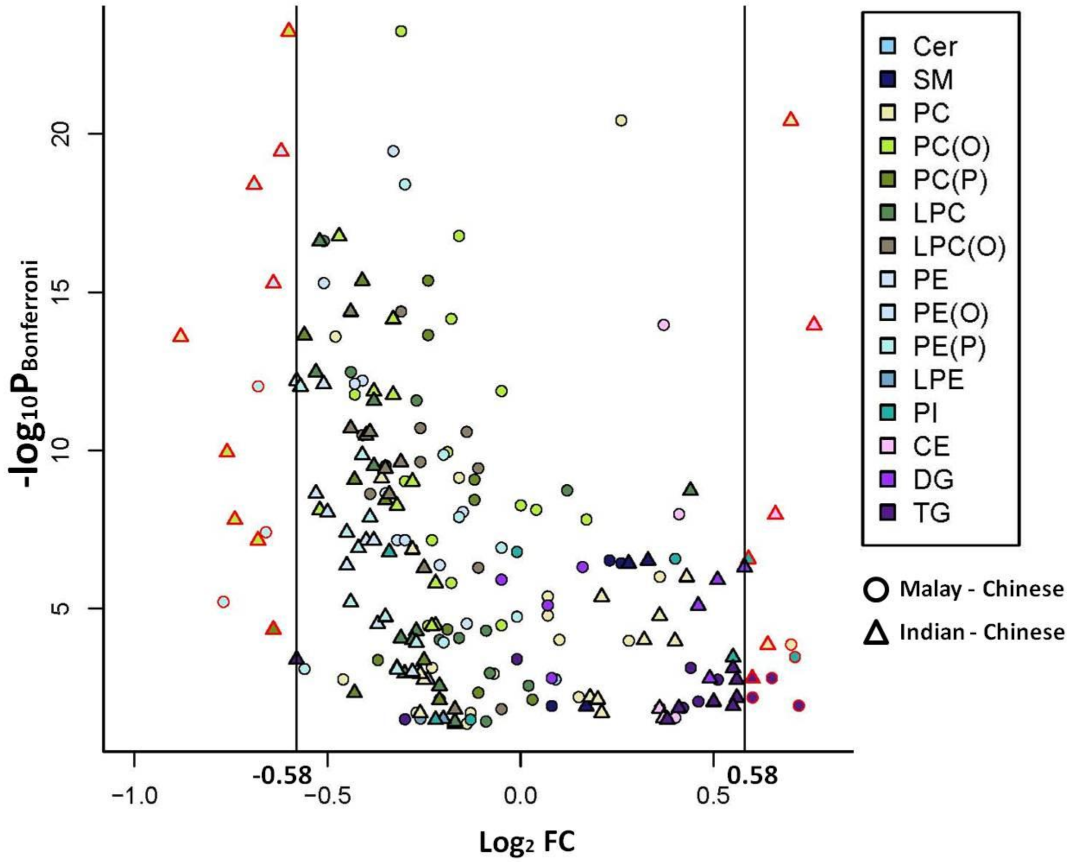

Supplementary 5. Distribution of the 107 significant lipid species with their fold-change, FC

Fold change was calculated with respect to Chinese, *i.e.* $\log_2$FC of <-0.58 and >0.58 correspond to at least 1.5 fold-change lower and higher respectively when compared against the Chinese. The *round* shape indicates the FC calculated between Malay and Chinese while the *triangular* shape indicates the FC calculated between Indian and Chinese. Shapes with red outline are representing lipid species with at least 1.5 fold-change. Each of the color represents each of the lipid classes. The details of the significant lipid species across 3 ethnic groups can be found in **Supplementary Data 4**.

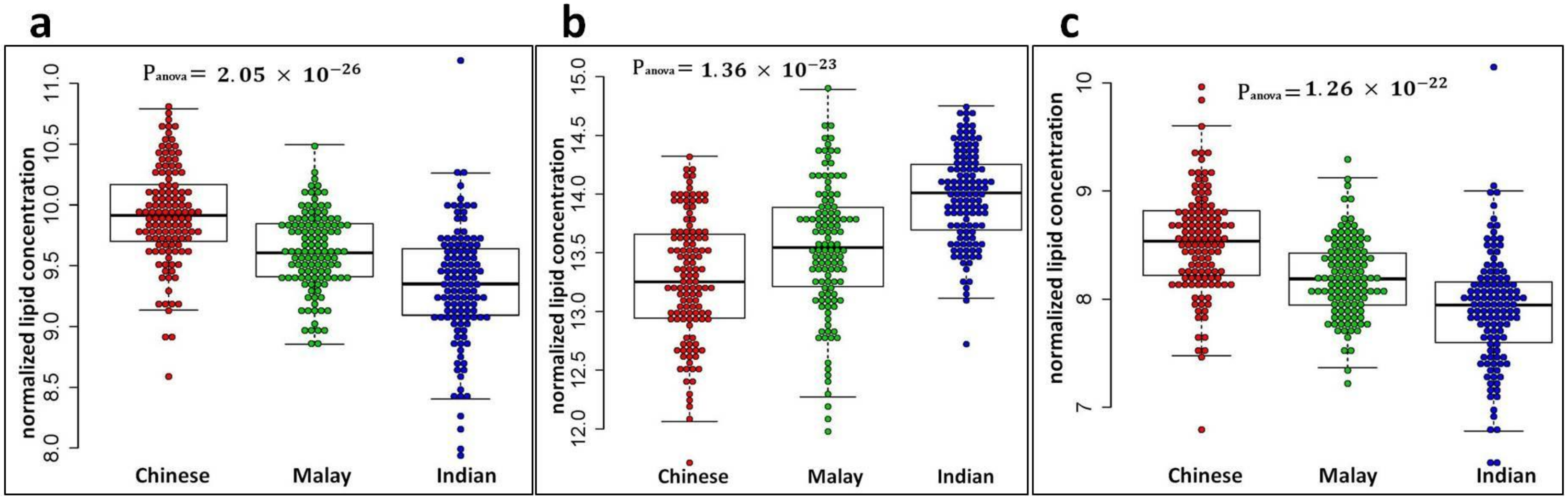

Supplementary Figure 6. A combined boxplot and scatter plot of the top three significant lipids across 3 ethnic groups. The combined plot of the distribution of lipid intensity of a)PC(O-40:7) b) PC_38:3 c) PE(O-40:7) across 3 ethnic groups. *P*-values were calculated using ANOVA, adjusted for gender and corrected for Bonferroni. The details of the significant lipids across 3 ethnic groups can be found in **Supplementary Data 4**.

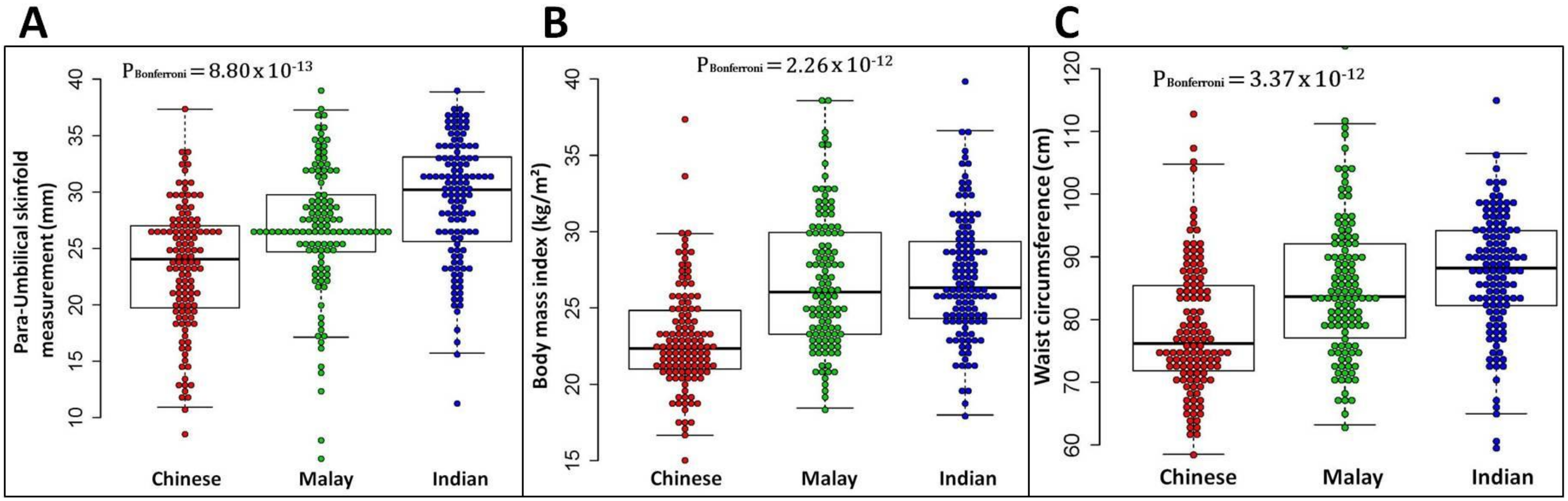

Supplementary Figure 7. A combined boxplot and scatter plot of the top three significant phenotypes across 3 ethnic groups. The combined plot of the distribution of a) Para-umbilical skinfold measurement b) BMI c) Waist circumference across 3 ethnic groups. *P-values* were calculated using ANOVA, adjusted for gender and corrected for Bonferroni. The details of the significant phenotypes across 3 ethnic groups can be found in **Supplementary Table 9.**

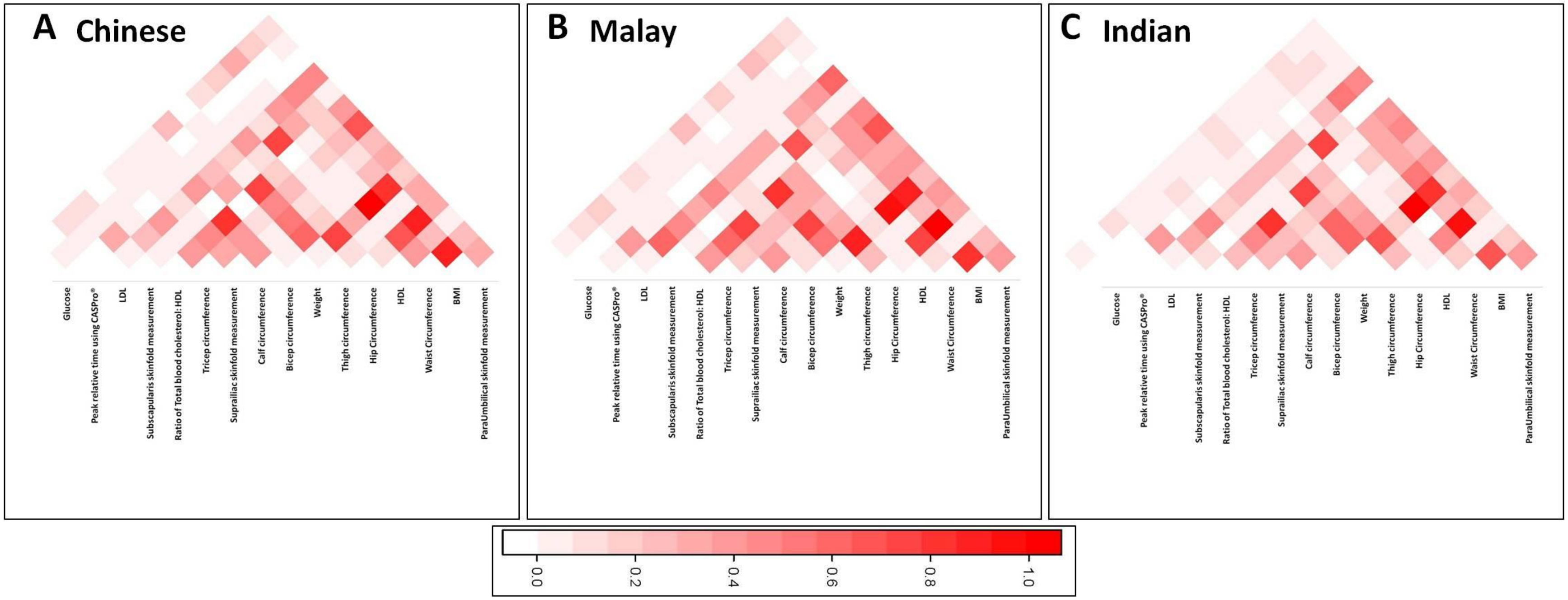

Supplementary Figure 8. Correlation of heatmap of the 16 significant phenotypes across 3 ethnic groups.
The correlation heatmap between the 16 significant phenotypes within **A)** Chinese; **B)** Malay; and **C)** Indian. The heatmap was calculated using Pearson's correlation, r^2. The intensity of the color reflects the magnitude of correlation, in which the white color means r^2=0 while red color means r^2=1.

Supplementary Tables

Supplementary Table 1. The corresponding whole genome sequencing IDs with IOMICS IDs in Malay and Indian population. The details of the whole genome sequencing has previously been describe[7,8].

IOMICS ID	Whole-Genome-Sequencing ID	Ethnic Group
M11051203	SSM030	Malay
M11050504	SSM100	Malay
M11051101	SSM010	Malay
M11050413	SSM007	Malay
M11050503	SSM058	Malay
M11050407	SSM027	Malay
M11051207	SSM094	Malay
M11050415	SSM098	Malay
M11051115	SSM079	Malay
M11050412	SSM038	Malay
M11050506	SSM097	Malay
M11051907	SSM083	Malay
M11051108	SSM055	Malay
M11051107	SSM061	Malay
M11050512	SSM019	Malay
M11050409	SSM078	Malay
M11050513	SSM059	Malay
M11072704	SSM047	Malay
M11050502	SSM036	Malay
M11050511	SSM063	Malay
M11050401	SSM082	Malay
M11051112	SSM064	Malay
M11051109	SSM068	Malay
M11050414	SSM056	Malay
M11050515	SSM022	Malay
M11050408	SSM003	Malay
M11050501	SSM033	Malay
M11050508	SSM009	Malay
M11050507	SSM012	Malay
M11050416	SSM071	Malay
M11050509	SSM011	Malay
M11062908	SSM035	Malay
M11050505	SSM015	Malay
M11060908	SSM029	Malay
M11051204	SSM051	Malay
M11050403	SSM042	Malay

M11052812	SSM017	Malay
M11051113	SSM053	Malay
M11050406	SSM099	Malay
M11050404	SSM057	Malay
M11051901	SSM040	Malay
M11051904	SSM075	Malay
M11050410	SSM074	Malay
M11051106	SSM006	Malay
M11072711	SSM092	Malay
M11062205	SSM002	Malay
M11051110	SSM028	Malay
M11051805	SSM026	Malay
M11050514	SSM069	Malay
M11051909	SSM077	Malay
M11051209	SSM081	Malay
M11050516	SSM073	Malay
M11051102	SSM008	Malay
M11051104	SSM084	Malay
M11050402	SSM095	Malay
M11050405	SSM054	Malay
M11051105	SSM080	Malay
M11051103	SSM067	Malay
M11052506	SSM023	Malay
M11050510	SSM066	Malay
M11051114	SSM085	Malay
M11050411	SSM044	Malay
M11060207	SSI020	Indian
M11062505	SSI028	Indian
M11061509	SSI032	Indian
M11062516	SSI031	Indian
M11060901	SSI013	Indian
M11062905	SSI039	Indian
M11060108	SSI018	Indian
M11060107	SSI021	Indian
M11062510	SSI026	Indian
M11052803	SSI019	Indian
M11060914	SSI033	Indian
M11062913	SSI005	Indian
M11060923	SSI035	Indian
M11060208	SSI025	Indian
M11061505	SSI038	Indian

M11060204	SSI030	Indian
M11062207	SSI024	Indian
M11062912	SSI017	Indian
M11062202	SSI023	Indian
M11070608	SSI015	Indian
M11061604	SSI011	Indian
M11062910	SSI040	Indian
M11052807	SSI022	Indian
M11060114	SSI008	Indian
M11071306	SSI003	Indian
M11062518	SSI029	Indian
M11062206	SSI010	Indian
M11060902	SSI009	Indian
M11060102	SSI036	Indian
M11060905	SSI006	Indian
M11052501	SSI014	Indian
M11062214	SSI034	Indian
M11062515	SSI037	Indian
M11061607	SSI004	Indian
M11062519	SSI027	Indian
M11070601	SSI012	Indian

Supplementary Table 2. The distribution of the sample size of Singapore integrative Omics cohort study in different technology platform.

**Omics**	**Chinese**	**Malay**	**Indian**	**Total**
Whole-Genome Sequencing	-	62	38	
Male	-	30	12	100
Female	-	32	26	
Genomics	110	108	105	
Male	44	38	38	323
Female	66	70	67	
HLA typing	111	119	120	
Male	44	42	43	350
Female	67	77	77	
Pharmacogenomics	106	112	115	
Male	41	40	39	333
Female	65	72	76	
Transcriptomics	98	75	96	
Male	37	35	38	269
Female	61	40	58	
Lipidomics	122	117	120	
Male	50	41	42	359
Female	72	76	78	
Non-coding RNAs(miRNAs)	117	115	119	
Male	47	42	41	351
Female	70	73	78	
**Non-Omics**				
Clinical,Lifestyle and Dietary	122	116	120	
Male	50	41	42	358
Female	72	75	78	

Supplementary Table 3. HLA alleles with Fst of ≥ 0.05 between 3 ethnic groups at each HLA locus.

		Frequency				
**Locus**	**Allele**	**Chinese**	**Malay**	**Indian**	**Wright Fst**	**Clinical PGx Implication^**
**A**	***24:07**	0.014	0.208	0.008	0.122	N.A
	***01:01**	0	0.008	0.142	0.089	N.A
**C**	***06:02**	0.023	0.013	0.161	0.074	N.A
	***07:04**	0	0.123	0.021	0.063	N.A
	***03:04**	0.162	0.038	0.021	0.058	N.A
**B**	***40:01**	0.208	0.043	0.025	0.081	(i) Patients with one or two copies of the HLA*40:01:01 allele have a decreased risk of severe cutaneous adverse reactions, such as Stevens-Johnson Syndrome and toxic epidermal necrolysis, when treated with carbamazepine as compared to patients with no HLA-B*40:01:01 alleles or negative for the HLA-B*40:01:01 test.
	***40:06**	0.009	0.009	0.138	0.075	N.A
	***46:01**	0.088	0.009	0	0.499	N.A
**DRB1**	***12:02**	0.106	0.390	0.03	0.166	N.A
	***09:01**	0.151	0.013	0	0.090	N.A
	***07:01**	0.018	0.102	0.198	0.057	N.A
**DQA1**	***06:01**	0.099	0.369	0.061	0.129	N.A
	***01:03**	0.061	0.036	0.263	0.097	N.A
	***03:01**	0.316	0.068	0.132	0.078	N.A
	***02:01**	0.014	0.104	0.184	0.053	N.A
**DQB1**	***03:01**	0.255	0.458	0.107	0.104	N.A
**DPA1**	***02:02**	0.614	0.270	0.108	0.200	N.A

	***01:03**	0.282	0.430	0.650	0.090	N.A
**DPB1**	***05:01**	0.436	0.164	0.029	0.173	N.A
	***04:01**	0.068	0.210	0.304	0.060	N.A

^Information were retrieved from PharmGKB®, only clinical implication with level 2a, 2b or 1 were retrieved.

Supplementary Table 4. Distribution of lipid species quantified in different lipid categories.

Lipid Category	Number of Species	^Sum(nmol/ml)
**Glycerophospholipids**		
Phosphatidylcholine - PC	50	805.93
Alkylphosphatidylcholine - PC(O)	18	24.87
Phosphatidylcholine plasmalogen - PC(P)	13	16.93
Lysophosphatidylcholine - LPC	21	87.74
Lysoalkylphosphatidylcholine - LPC(O)	9	0.96
Phosphatidylethanolamine - PE	17	47.61
Alkylphosphatidylethanolamine - PE(O)	10	5.17
Phosphatidylethanolamine plasmalogen - PE(P)	11	52.89
Lysophosphatidylethanolamine - LPE	6	6.00
Phosphatidylinositol - PI	15	47.08
Total	170	1095.20
**Sphingolipids**		
Ceramide - Cer	10	9.70
Monohexosylceramide - HexCer	6	11.86
Trihexosylceramide - Hex3Cer	1	1.91
GM_3 ganglioside - GM3	1	1.43
Sphingomyelin - SM	21	257.93
Total	39	282.83
**Sterol Lipids**		
Cholesterol Esters - CE	17	4103.86
Cholesterol - COH	1	2246.03
Total	18	6349.89
**Glyecerolipids**		
Diglycerides - DG	18	59.19
Triglycerides - TG	37	565.55
Total	55	624.74
**Total**	282	8352.66

^ sum of the average of the lipid species of 359 individuals (pmol/ml has converted to nmol/ml) in each lipid category

Supplementary Table 5. 29 Tagging and 42 Tagged lipids in the Chinese from the 125 high correlated (r^2>*0.8*) lipid pairs. The lipids in bold are identified to be most differentiated lipid across 3 ethnic groups.

Tagging	Tagged
Cer(d18:0/22:0)	Cer(d18:0/24:0)
PE(34:1)	PE(36:1)
Cer(d18:0/24:1)	Cer(d18:1/24:0)
PC(34:5)	PC(36:6)
HexCer(d18:1/22:0)	HexCer(d18:1/24:0)
**PC(16:0_20:4)**	LPC(20:5)
**PC(O-32:2)**	**PC(O-34:3)**
PE(36:2)	PE(34:2), PE(36:3)
DG(16:0_18:1)	DG(18:1_18:1), TG(16:0_18:1_18:1)
**DG(16:0_20:4)**	DG(18:1_20:4)
**PE(O-36:4)**	**PE(O-38:5)**
**TG(14:0_16:0_18:1)**	**TG(14:0_16:0_18:2)**, TG(16:1_16:1_18:0), TG(14:0_18:0_18:1)
TG(18:0_18:0_18:1)	DG(18:0_18:1), TG(16:0_18:0_18:1), TG(18:0_18:1_18:1)
PC(O-34:2)	PC(P-34:1)
**TG(15:0_16:0_18:1)**	TG(16:0_17:0_18:2), TG(16:0_17:0_18:1), **TG(14:0_17:0_18:1)**, **TG(16:0_16:1_17:0)**
**PC(O-36:5)**	**PC(P-36:5)**
PE(38:4)	PE(40:4)
TG(16:1_17:0_18:1)	TG(15:0_18:1_18:1), TG(17:0_18:1_18:1)
TG(16:1_16:1_18:1)	DG(16:1_18:1), TG(14:1_16:1_18:0), TG(16:0_16:1_18:1), TG(16:1_16:1_16:1)
**DG(18:1_20:3)**	**DG(18:2_20:3)**
DG(18:1_18:2)	DG(18:2_18:2)
**PE(P-16:0/18:2)**	**PE(P-18:0/18:2)**
TG(14:0_16:1_18:2)	TG(14:0_16:1_18:1)
TG(14:0_18:2_18:2)	TG(14:1_18:1_18:1)

TG(16:0_18:1_18:2)	TG(16:0_18:2_18:2)
DG(16:0_18:2)	DG(18:0_18:2)
DG(18:2_20:4)	**TG(18:2_18:2_20:4)**
**TG(16:0_16:0_18:1)**	TG(16:0_16:0_18:2)
TG(16:1_18:1_18:2)	TG(18:1_18:2_18:2)

Supplementary Table 6. 26 Tagging and 35 Tagged lipids in the Malays from the 125 high correlated (r^2>*0.8*) lipid pairs. The lipids in bold are identified to be most differentiated lipid across 3 ethnic groups.

Tagging	Tagged
Cer(d18:0/22:0)	Cer(d18:0/24:0)
**TG(16:0_16:0_18:1)**	TG(16:0_18:0_18:1), TG(16:0_16:0_18:2)
Cer(d18:0/24:1)	Cer(d18:1/24:0)
**PC(16:0_20:4)**	LPC(20:5)
**PC(29:0)**	**PC(30:0)**
**DG(18:1_20:3)**	**DG(18:2_20:3)**
PE(34:2)	PE(36:2), PE(36:3)
PE(38:6)	PE(40:6)
PC(O-34:2)	PC(P-34:1)
**TG(15:0_16:0_18:1)**	**TG(14:0_17:0_18:1)**,**TG(16:0_16:1_17:0)**, TG(16:0_17:0_18:1)
SM(35:1)	SM(35:2)
DG(18:1_18:2)	DG(18:2_18:2)
**PC(O-36:5)**	**PC(P-36:5)**
TG(16:0_18:2_18:2)	TG(18:0_18:2_18:2), TG(18:1_18:2_18:2)
**TG(14:0_16:0_18:1)**	TG(16:1_16:1_18:0), TG(14:0_18:0_18:1), **TG(14:0_16:0_18:2)**
TG(16:1_16:1_18:1)	DG(16:1_18:1), TG(16:1_16:1_16:1)
TG(18:0_18:1_18:1)	TG(18:0_18:0_18:1)
DG(18:1_18:1)	TG(16:0_18:1_18:1)
TG(14:0_16:1_18:2)	TG(14:0_16:1_18:1)
**DG(16:0_20:3)**	**DG(16:0_20:4)**
DG(16:0_18:1)	DG(18:0_18:1)
DG(16:0_18:2)	DG(18:0_18:2)
TG(14:1_16:1_18:0)	**TG(14:1_16:0_18:1)**, TG(16:0_16:1_18:1)
DG(18:1_20:4)	DG(18:2_20:4)
TG(16:0_17:0_18:2)	TG(16:1_17:0_18:1)
TG(15:0_18:1_18:1)	TG(17:0_18:1_18:1)

Supplementary Table 7. 26 Tagging and 34 Tagged lipids in the Indians from the 65 high correlated (r^2*>0.8*) lipid pairs. The lipids in bold are identified to be most differentiated lipid across 3 ethnic groups.

Tagging	Tagged
Cer(d18:0/22:0)	Cer(d18:0/24:0)
TG(16:1_16:1_18:0)	**TG(14:0_16:0_18:1)**, **TG(14:0_16:0_18:2)**,TG(14:0_18:0_18:1)
Cer(d18:0/24:1)	Cer(d18:1/24:0)
**PC(37:6)**	**PC(39:6)**
HexCer(d18:1/22:0)	HexCer(d18:1/24:0)
**PC(O-32:2)**	**PC(O-34:4)**
**PC(16:0_20:4)**	LPC(20:5)
PE(34:2)	PE(36:2)
TG(16:0_18:1_18:2)	TG(16:0_18:2_18:2)
**PE(P-16:0/20:4)**	**PE(P-18:0/20:4)**
PC(38:7)	PC(40:6)
**PE(O-36:4)**	**PE(O-38:4)**
**PE(P-16:0/22:6)**	**PE(P-18:0/22:6)**
PI(38:6)	PI(40:6)
TG(16:0_16:1_18:1)	TG(14:1_16:1_18:0),TG(16:1_16:1_18:1)
PC(O-34:2)	PC(P-34:1)
**TG(15:0_16:0_18:1)**	**TG(16:0_16:1_17:0)**, **TG(14:0_17:0_18:1)**, TG(16:0_17:0_18:1), TG(16:0_17:0_18:2)
**PC(O-36:5)**	**PC(P-36:5)**
DG(16:0_18:1)	DG(18:0_18:1)
PE(38:6)	PE(40:6)
TG(14:0_16:1_18:1)	TG(14:0_16:1_18:2)
**TG(16:0_16:0_18:1)**	TG(16:0_16:0_18:2)
DG(16:0_18:2)	DG(18:0_18:2)
TG(16:1_17:0_18:1)	DG(18:1_18:3)
**DG(16:0_20:4)**	TG(15:0_18:1_18:1), TG(17:0_18:1_18:1)
DG(18:1_18:1)	DG(18:1_20:4)
TG(16:0_18:0_18:1)	TG(18:0_18:0_18:1)

Supplementary Table 8 Of the 107 differentially expressed lipids across 3 ethnic groups, following is the list of 98, 99 and 97 tagging lipids in the Chinese, Malays and Indians respectively.

Chinese	Malay	Indian
Cer(d18:1/24:1)	Cer(d18:1/24:1)	Cer(d18:1/24:1)
SM(32:0)	SM(32:0)	SM(32:0)
SM(32:2)	SM(32:2)	SM(32:2)
SM(41:1)	SM(41:1)	SM(41:1)
PC(28:0)	PC(28:0)	PC(28:0)
PC(29:0)	PC(29:0)	PC(29:0)
PC(30:0)	PC(31:0)	PC(30:0)
PC(31:0)	PC(32:3)	PC(31:0)
PC(32:3)	PC(34:4)	PC(32:3)
PC(34:4)	PC(35:2)	PC(34:4)
PC(35:2)	PC(36:0)	PC(35:2)
PC(36:0)	PC(36:3)	PC(36:0)
PC(36:3)	PC(16:0_20:4)	PC(36:3)
PC(16:0_20:4)	PC(37:6)	PC(16:0_20:4)
PC(37:6)	PC(38:3)	PC(37:6)
PC(38:3)	PC(38:4)	PC(38:3)
PC(38:4)	PC(16:0_22:6)	PC(38:4)
PC(16:0_22:6)	PC(39:6)	PC(16:0_22:6)
PC(39:6)	PC(39:7)	PC(39:7)
PC(39:7)	PC(40:7)	PC(40:7)
PC(40:7)	PC(40:8)	PC(40:8)
PC(40:8)	PC(O-32:1)	PC(O-32:1)
PC(O-32:1)	PC(O-32:2)	PC(O-32:2)
PC(O-32:2)	PC(O-34:3)	PC(O-34:3)
PC(O-34:4)	PC(O-34:4)	PC(O-36:1)
PC(O-36:1)	PC(O-36:1)	PC(O-36:2)
PC(O-36:2)	PC(O-36:2)	PC(O-36:3)
PC(O-36:3)	PC(O-36:3)	PC(O-36:4)
PC(O-36:4)	PC(O-36:4)	PC(O-36:5)
PC(O-36:5)	PC(O-36:5)	PC(O-38:4)
PC(O-38:4)	PC(O-38:4)	PC(O-38:5)
PC(O-38:5)	PC(O-38:5)	PC(O-40:5)
PC(O-40:5)	PC(O-40:5)	PC(O-40:6)
PC(O-40:6)	PC(O-40:6)	PC(O-40:7)
PC(O-40:7)	PC(O-40:7)	PC(P-32:1)

PC(P-32:1)	PC(P-32:1)	PC(P-34:3)
PC(P-34:3)	PC(P-34:3)	PC(P-36:2)
PC(P-36:2)	PC(P-36:2)	PC(P-38:4)
PC(P-38:4)	PC(P-38:4)	PC(P-38:5)
PC(P-38:5)	PC(P-38:5)	PC(P-38:6)
PC(P-38:6)	PC(P-38:6)	PC(P-40:6)
PC(P-40:6)	PC(P-40:6)	LPC(17:1)
LPC(17:1)	LPC(17:1)	LPC(18:0)
LPC(18:0)	LPC(18:0)	LPC(18:1)
LPC(18:1)	LPC(18:1)	LPC(18:2)
LPC(18:2)	LPC(18:2)	LPC(20:0)
LPC(20:0)	LPC(20:0)	LPC(20:1)
LPC(20:1)	LPC(20:1)	LPC(20:2)
LPC(20:2)	LPC(20:2)	LPC(20:3)
LPC(20:3)	LPC(20:3)	LPC(22:0)
LPC(22:0)	LPC(22:0)	LPC(22:1)
LPC(22:1)	LPC(22:1)	LPC(22:6)
LPC(22:6)	LPC(22:6)	LPC(O-16:0)
LPC(O-16:0)	LPC(O-16:0)	LPC(O-18:0)
LPC(O-18:0)	LPC(O-18:0)	LPC(O-18:1)
LPC(O-18:1)	LPC(O-18:1)	LPC(O-20:1)
LPC(O-20:1)	LPC(O-20:1)	LPC(O-22:0)
LPC(O-22:0)	LPC(O-22:0)	LPC(O-22:1)
LPC(O-22:1)	LPC(O-22:1)	LPC(O-24:0)
LPC(O-24:0)	LPC(O-24:0)	LPC(O-24:1)
LPC(O-24:1)	LPC(O-24:1)	LPC(O-24:2)
LPC(O-24:2)	LPC(O-24:2)	PE(40:7)
PE(40:7)	PE(40:7)	PE(O-34:1)
PE(O-34:1)	PE(O-34:1)	PE(O-34:2)
PE(O-34:2)	PE(O-34:2)	PE(O-36:2)
PE(O-36:2)	PE(O-36:2)	PE(O-36:3)
PE(O-36:3)	PE(O-36:3)	PE(O-36:4)
PE(O-36:4)	PE(O-36:4)	PE(O-38:5)
PE(O-38:4)	PE(O-38:4)	PE(O-40:5)
PE(O-40:5)	PE(O-38:5)	PE(O-40:6)
PE(O-40:6)	PE(O-40:5)	PE(O-40:7)
PE(O-40:7)	PE(O-40:6)	PE(P-16:0/18:1)
PE(P-16:0/18:1)	PE(O-40:7)	PE(P-16:0/18:2)
PE(P-16:0/18:2)	PE(P-16:0/18:1)	PE(P-16:0/20:4)

PE(P-16:0/20:4)	PE(P-16:0/18:2)	PE(P-16:0/22:6)
PE(P-16:0/22:6)	PE(P-16:0/20:4)	PE(P-18:0/18:1)
PE(P-18:0/18:1)	PE(P-16:0/22:6)	PE(P-18:0/18:2)
PE(P-18:0/20:4)	PE(P-18:0/18:1)	PE(P-18:0/22:5)
PE(P-18:0/22:5)	PE(P-18:0/18:2)	PE(P-20:0/20:4)
PE(P-18:0/22:6)	PE(P-18:0/20:4)	LPE(18:0)
PE(P-20:0/20:4)	PE(P-18:0/22:5)	PI(32:0)
LPE(18:0)	PE(P-18:0/22:6)	PI(32:1)
PI(32:0)	PE(P-20:0/20:4)	PI(38:2)
PI(32:1)	LPE(18:0)	PI(40:4)
PI(38:2)	PI(32:0)	CE(14:0)
PI(40:4)	PI(32:1)	CE(16:2)
CE(14:0)	PI(38:2)	CE(18:3)
CE(16:2)	PI(40:4)	CE(20:3)
CE(18:3)	CE(14:0)	DG(16:0_20:3)
CE(20:3)	CE(16:2)	DG(16:0_20:4)
DG(16:0_20:3)	CE(18:3)	DG(18:1_20:3)
DG(16:0_20:4)	CE(20:3)	DG(18:2_20:3)
DG(18:1_20:3)	DG(16:0_20:3)	TG(14:1_16:0_18:1)
TG(14:0_16:0_18:1)	DG(18:1_20:3)	TG(15:0_16:0_18:1)
TG(14:1_16:0_18:1)	TG(14:0_16:0_18:1)	TG(16:0_16:0_18:1)
TG(15:0_16:0_18:1)	TG(15:0_16:0_18:1)	TG(18:1_18:1_22:6)
TG(16:0_16:0_18:1)	TG(16:0_16:0_18:1)	TG(18:2_18:2_20:4)
TG(18:1_18:1_22:6)	TG(18:1_18:1_22:6)	-
-	TG(18:2_18:2_20:4)	-

Supplementary Table 9. 16 most differentiated phenotypes between 3 ethnic groups after adjusted for gender effect. Non-parametric test, *Kruskal-Wallis* was also performed for the 16 phenotypes.

Phenotype	$P_{bonferroni}$	$P_{kruskal}$	Chinese (mean±SE)	Malay (mean±SE)	Indian (mean±SE)
ParaUmbilical skinfold measurement (mm)	8.80E-13	1.51E-13	23.4±0.50	26.7±0.53	29.2±0.50
BMI (kg/m²)	2.26E-12	3.28E-17	23.0±0.31	26.7±0.41	27.0±0.37
Waist Circumsference (cm)	3.37E-12	1.40E-11	78.4±0.92	85.0±1.03	87.4±0.88
HDL (mmol/L)	1.99E-11	3.62E-09	1.48±0.04	1.35±0.03	1.19±0.02
Hip Circumsference (cm)	6.67E-11	1.82E-15	94.1±0.58	100.2±0.79	101.5±0.75
Thigh circumsference (mm)	1.37E-10	1.35E-10	21.0±0.58	26.7±0.71	26.8±0.67
Weight (kg)	2.65E-10	2.23E-10	60.0±1.05	68.0±1.23	69.5±1.04
Bicep circumsference (mm)	2.26E-08	7.72E-10	9.47±0.29	12.6±0.46	13.0±0.48
Calf circumsference (mm)	2.80E-08	2.92E-12	14.5±0.36	19.7±0.62	19.2±0.57
Suprailiac skinfold measurement (mm)	2.23E-07	1.53E-07	21.7±0.55	24.8±0.56	26.1±0.45
Tricep circumsference (mm)	4.80E-07	3.01E-12	15.8±0.41	21.2±0.55	19.7±0.51
Ratio of Total blood cholesterol: HDL	1.54E-06	6.84E-07	4.11±0.12	4.60±0.12	4.87±0.10
Subscapularis skinfold measurement (mm)	2.37E-05	1.08E-08	19.7±0.56	24.3±0.69	24.1±0.56
LDL (mmol/L)	2.22E-03	4.52E-05	3.50±0.07	3.92±0.08	3.92±0.07
Peak relative time using CASPro® (milisecond)	2.22E-02	2.61E-03	106.6±2.21	99.4±2.01	95.8±2.14
Glucose (mmol/L)	4.74E-02	3.07E-02	4.98±0.04	5.21±0.07	5.24±0.07

Supplementary Table 10. List of the food items with loadings *cut-off > 0.070* and *<-0.070* at the first PC from the principal component analysis (**Figure 1F**). As the sum of squares of all loadings for an individual principal component is one, we can calculate what is the loadings would be if all variables contributed equally to that principal component. Hence, the loadings cut-off is calculated as squared root of 1/number of variables.

**PC1**		
**Food group**	**Food Item**	**loadings**
Legumes_95	Dried legumes (eg. Dhal, dried beans) in gravy	0.115
Fish_255	Fish Curry without coconut	0.110
poultry_150	Poultry Curry without coconut	0.107
otherBread_N3	Dosai/Thosai	0.102
otherBread_N2	Chapati	0.090
OtherDesserts_301	Dim sum-steamed (eg. Chee cheong fun, dumplings, rice dumplings)	-0.179
MeatLean_200	Roasted/grilled/BBQ lean meat	-0.177
dryNoodles_27	Fishball/yong tau foo/wanton/minced meat & mushrooms/prawn/beef/chicken	-0.171
MeatLeanFat_225	Roasted/grilled/BBQ lean meat and meat with fat	-0.170
noodlesInGravy_33	Laksa lemak (incl. laksa noodle and lontong)	-0.167
Fish_243	steamed fish	-0.163
friedNoodles_30	Fried hor fun (incl. all noodles fried with starchy gravy, may be added with meat or seafood)	-0.158
PoultryWithSkin_159	Stir fried poultry with skin	-0.156
others_125	soups with meat stock	-0.152
MeatLeanFat_205	Stir fried lean meat and meat with fat	-0.150
PoultryWithSkin_179	Steamed poultry with skin	-0.150
soups_601	Clear Soup/broth	-0.141
PoultryWithSkin_175	Stew/braised/roasted poultry with skin	-0.138
MeatPreserved_231	Ham	-0.138
OtherDesserts_302	Dim sum-fried/deep fried(eg. Fried carrot cake, wanton, char siew puff)	-0.137
MeatLean_180	Stir fried lean meat	-0.136
MeatLean_204	Steamed/soup with lean meat	-0.135

DessertsInSoup_297	Soupy dessert without coconut milk (eg. Cheng teng, green bean soup, tau suan)	-0.133
friedNoodles_29	Fried kway teow with cockles	-0.130
MeatPreserved_233	Canned(eg.luncheon meat, corned beef)	-0.127
MeatLean_196	Stew/braised lean meat	-0.127
flavoredRice_18	Chicken/duck rice (with and without skin)	-0.127
OtherSeafood_287	Grilled seafood	-0.126
riceAndPorridge_16	Plain rice porridge (white, brown, or red)	-0.125
KuehKueh_299	Kueh without coconut milk (eg. Kueh tutu, soon kway)	-0.121
noodle_25	Fishball/yong tau foo/wanton/minced meat & mushrooms/prawn/beef/chicken	-0.120
OtherSeafood_263	stir fried/ pan fried/ deep fried seafood	-0.114
MeatLeanFat_221	Stew/braised/roasted lean meat and meat with fat	-0.108
CannedVege_704	Preserved vegetables (Chye Sim, Olives, Kimchi etc.)	-0.106
Biscuits_308	Sponge cake	-0.106
friedNoodles_32	Fried beehoon(fried dry beehoon)	-0.105
OtherSeafood_271	steamed seafood	-0.105
FastFood_1100	Mashed potato with gravy	-0.104
DarkGreenVege_61	Stir fried dark green vegetables in oyster sauce	-0.101
MeatLeanFat_229	Steamed/soup lean meat and meat with fat	-0.100
Fish_3003	Raw fish (eg. Sashimi)	-0.097
flavoredRice_22	Claypot rice	-0.095
Biscuits_307	Plain butter cake/fruit cake	-0.094
flavoredRice_19	Mui fan	-0.088
flavoredRice_24	Flavoured porridge (e.g. chicken, pork, duck, fish, peanut, century egg)	-0.087
FastFood_310	Burgers, with beef or chicken	-0.087
MeatPreserved_232	Bacon	-0.083
MeatPreserved_234	Liver and other innards (incl. kway chap without egg and kway)	-0.082
Tea_N25	Chinese tea(brewed)	-0.079
Titbits_318	Ice-cream	-0.078
soups_600	Cream soup	-0.077

flavoredRice_23	Glutinous rice (incl. lo mai khai, lotus leaf rice, rice dumplings)	-0.076
PoultryWithSkin_167	coconut curry	-0.076
MeatLeanFat_209	Pan/deep fried	-0.075
fruit_133	Orange/red/yellow fresh fruits	-0.075
mixedVegetables_107	Stir fried mixed vegetables in oyster sauce	-0.071
Vegetarian_401	Gluten(char siew/duck)	-0.071

Supplementary Table 11. List of the food items with loadings *cut-off > 0.070* and *<-0.070* at the second PC from the principal component analysis (**Figure 1F**).

**PC2**		
**Food group**	**Food Item**	**loadings**
soups_601	Clear Soup/broth	0.153
Fish_243	steamed fish	0.133
others_125	soups with meat stock	0.126
DarkGreenVege_69	Raw/steamed/in soup dark green vegetables	0.115
PaleGreenVege_52	Raw/steamed/in soup pale green vegetables	0.102
noodle_25	Fishball/yong tau foo/wanton/minced meat & mushrooms/prawn/beef/chicken	0.100
MeatLean_204	Steamed lean meat/lean meat in soup	0.099
tomatoes_82	Raw tomatoes /steamed tomatoes/tomatoes in soup	0.085
MeatLean_180	Stir fried lean meat	0.082
poultry_158	steamed poultry	0.081
mixedVegetables_115	Raw mixed vegetables /steamed mixed vegetables/mixed vegetables in soup/Chinese rojak	0.075
MeatLean_196	Stew/braised lean meat	0.074
Biscuits_306	Puff/flaky pastries(croissants,baked,curry puffs etc)	-0.203
FastFood_311	Burgers, Fish	-0.195
FastFood_313	Pizza	-0.188
flavoredRice_20	Nasi briyani	-0.173
FastFood_1100	Mashed potato with gravy	-0.159
Biscuits_307	Plain butter cake/fruit cake	-0.158
friedNoodles_31	Fried noodles (incl. hokkien mee, mee goreng)	-0.156
flavoredRice_21	Nasi Lemak	-0.141
Titbits_319	Chocolate	-0.135
PoultryWithSkin_163	Pan/deep fried poultry with skin	-0.133
friedNoodles_29	Fried kway teow with cockles	-0.131

OtherSeafood_283	seafood curry without coconut	-0.130
noodlesInGravy_34	mee siam	-0.122
DessertsInSoup_296	Soupy dessert with coconut milk/cream(eg. Pulor hitam, buborcha cha)	-0.122
OtherDesserts_303	sweet indian snacks(eg. Burfi, halwa)	-0.117
FastFood_312	French fries	-0.117
FastFood_310	Burgers, with beef or chicken	-0.116
noodlesInGravy_28	lor mee/ mee rebus	-0.115
Biscuits_309	cream cakes	-0.114
Fish_247	Assam pedas fish	-0.112
KuehKueh_298	Steamed kueh with coconut/coconut milk/coconut cream(eg kueh sarlat, kueh dadar, putu mayam,idli)	-0.111
PlainCoffee_N22	Gourmet coffee(mocha, frappuccino, cappuccino)	-0.109
MeatLean_188	lean meat in coconut curry/rendang	-0.107
OtherDesserts_300	Fried snacks (eg. You tiao, goreng pisang, Indian rojak)	-0.107
otherNoodles_907	Boiled noodles/spaghetti/pasta with cream white sauce	-0.107
BeverageVegeFruit_N12	Sugared fruit drinks/other sugared drinks made from syrup/cordial (eg. Lemonade, Roohafza)/other sweetened canned or packed drinks	-0.106
Titbits_317	Fried salty snacks (crisps, prawn crackers, keropok, salted biscuits etc)	-0.106
Titbits_318	Ice-cream	-0.104
flavoredRice_18	Chicken/duck rice (with and without skin)	-0.101
flavoredRice_17	Fried rice	-0.099
BeverageVegeFruit_N10	Other fruit juices (100%)	-0.095
OtherSeafood_267	Deep fried with batter	-0.092
MeatPreserved_234	Liver and other innards (incl. kway chap without egg and kway)	-0.092
PlainCoffee_N21	sweetened bottled/canned coffee drinks	-0.088
otherBread_9	Roti prata/murtabak	-0.087
otherBread_11	French toast/roti telur/ roti john	-0.086
poultry_150	Poultry curry without coconut	-0.086
flavoredRice_19	Mui fan	-0.085

MeatLean_192	Lean meat curry without coconut	-0.082
BeverageSoftDrinks_N16	Other carbonated drinks with sugar but non-caffeinated(eg. 7-up, root beer)	-0.081
otherBread_N3	Dosai/Thosai	-0.078
SoyaProducts_1201	soya beancurd (tau huay)	-0.077
others_121	vegetable stems or roots in curry lemak	-0.075
DarkGreenVege_65	Stir fried dark green vegetables in sambal belacan/dried prawns	-0.075
MeatPreserved_230	Sausages	-0.075
WholeEggs_292	Fried/scrambled whole eggs	-0.074
Legumes_95	Dried legumes (eg. Dhal, dried beans) in gravy	-0.073
PartA_BeverageSoftDrinks_N14	other carbonated low calorie drinks without caffeine (diet 7-up)	-0.072
PartA_BeverageSoftDrinks_N15	Carbonated drinks with sugar and caffeine(Coca-cola,pepsi)	-0.071

Supplementary Table 12. List of the food items with loadings *cut-off > 0.070* and *<-0.070* at the third PC from the principal component analysis (**Supplementary Figure 1A**).

**PC3**		
**Food group**	**Food Item**	**loadings**
Fish_247	Assam pedas fish	0.149
Fish_235	Stir fried/pan fried/deep fried fish	0.129
MeatLean_188	Lean meat in coconut curry/rendang	0.118
flavoredRice_21	nasi lemak	0.103
DarkGreenVege_65	Stir fried dark green vegetables in sambal belacan/dried prawns	0.093
noodlesInGravy_28	lor mee/ mee rebus	0.089
PlainCoffee_N20	Instant 2-in-1 or 3-in-1 coffee	0.089
OtherDesserts_300	Fried snacks (eg. You tiao, goreng pisang, Indian rojak)	0.088
Fish_251	Fish curry with coconut	0.088
MeatLeanFat_213	Lean meat and meat with fat in coconut curry/rendang	0.087
riceAndPorridge_15	Plain rice (white, brown or red)	0.085
MeatPreserved_234	Liver and other innards (incl. kway chap without egg and kway)	0.083
PoultryWithSkin_167	Poultry with skin in coconut curry	0.083
PoultryWithSkin_163	Pan/deep fried poultry with skin	0.083
PoultryWithSkin_175	Stew/braised/roasted poultry with skin	0.082
PoultryWithSkin_179	Steamed poultry with skin	0.078
otherNoodles_35	Instand noodles	0.074
Legumes_95	Dried legumes (eg. Dhal, dried beans) in gravy	-0.206
otherBread_N4	Breads made from other flour(rye, pearl millet (bajra), sorghum (jowar), or finger millets (raji)]	-0.204
Nuts_315	Dried/roasted nuts	-0.192
Cereals_14	Mixed cereals with fruits/nuts/breakfast cereals	-0.181
breads_2	Wholemeal bread	-0.177
poultry_150	Poultry Curry without coconut	-0.173

Vegetarian_402	Fried beancurd sheet	-0.159
Vegetarian_400	Fried vegetarian kway teow/beehoon/mee/rice	-0.155
Cheese_347	Low fat cheese	-0.153
otherNoodles_906	Boiled noodle/spaghetti/pasta with tomato sauce	-0.150
otherBread_N2	Chapatti	-0.141
Yoghurt_344	Regular	-0.141
others_121	vegetable stems or roots in curry lemak	-0.139
mixedVegetables_111	Mixed vegetables in curry Lemak	-0.133
Fish_255	Fish curry without coconut	-0.131
saladDressing_132	Oil-based dressing salad dressing(olive oil, Italian dressing)	-0.131
OtherSeafood_283	Seafood curry without coconut	-0.130
Legumes_354	Raw/steamed/boiled legumes	-0.129
otherBread_N3	Dosai/Thosai	-0.128
tomatoes_70	Stir fried tomatoes, plain	-0.119
poultry_154	Stew/braised/roasted poultry	-0.118
tomatoes_78	tomatoes in curry/lemak	-0.117
Yoghurt_345	low fat(including frozen yoghurt	-0.112
Legumes_83	Stir fried legumes, plain	-0.112
Vegetarian_401	Gluten(char siew/duck)	-0.110
OtherDesserts_303	Sweet Indian snacks(eg burfi,halwa)	-0.109
tomatoes_82	Raw tomatoes/steamed tomatoes/tomatoes in soup	-0.108
BeverageVegeFruit_N9	Grapefruit juice	-0.099
Titbits_319	Chocolate	-0.093
otherNoodles_907	Boiled noodles/spaghetti/pasta with cream white sauce	-0.091
Malt_1320	Malt beverages (e.g. hot chocolate, horlicks®,Milo®,Ovaltine®)	-0.088
PlainCoffee_N19	Instant Coffees (exclude 2-in-1/3-in-1)	-0.087
Fish_3003	Raw fish(eg. Sashimi)	-0.083
mixedVegetables_115	Raw mixed vegetables/steamed mixed vegetables/mixed vegetables in soup/Chinese rojak	-0.081
Tea_N26	Green Tea (brewed)	-0.081

MeatPreserved_231	Ham	-0.078
fruit_134	Other fresh fruits	-0.078
Tea_N23	Sweetened bottled tea (non-brewed, eg ice lemon/peach teas)	-0.075
MilkAsDrink_342	Low fat milk (fresh/UHT/powder)	-0.075
PaleGreenVege_48	pale green vegetables in Curry/lemak	-0.074
saladDressing_131	Creamy salad dressing-light/low fat	-0.073
Fish_259	Grilled fish	-0.072

Supplementary Table 13. List of internal standards (ISTDs) and their concentrations used in this study

**Name**	**pmol/sample**
BMP 14:0 14:0	100
Ceramide (Cer) 17:0	100
Ceramide-1-P (Cer-1-P) 12:0	100
1-O-Acyl-Ceramide	100
Dihydroceramide (dhCer) 8:0	50
Galactosylceramide (GalCer) 15:0	100
Sphingomyelin (SM) 12:0	200
Sphingosine (Sph) 17:1 base	100
Sphingosine-1-phosphate (Sph-1-P) 17:1 base	100
Sphinganine (Sphn) 17:0 base	100
Sphinganine-1-Phosphate (Sphn-1-P) 17:0 base	100
Phosphatidic Acid (PA) 17:0 17:0	100
Phosphatidylcholine (PC) 13:0 13:0	100
Phosphatidylethanolamine (PE) 17:0 17:0	100
Phosphatidylglycerol (PG) 17:0 17:0	100
Phosphatidylserine (PS) 17:0 17:0	100
Lysophosphatidic acid (LPA) 17:0	100
Lysophosphatidylcholine (LPC) 13:0	100
Diacylglycerol (DAG) 15:0 15:0	200
Glyceryl triheptadecanoate (TAG) 17:0 17:0 17:0	100
Cholesterol (D7)	10000
CE 18:0 (d6)	1000
LysoPE 14:0	100
Cardiolipin 14:0	100
Glucosylceramide 16:0 d3	50
Lactosylceramide 16:0 d3	50
Trihexosylceramide 17:0	50
Lysophosphatidylcholine (LPC) 19:0	100

Supplementary References

1 Alshehry, Z. H. *et al.* An Efficient Single Phase Method for the Extraction of Plasma Lipids. *Metabolites* **5**, 389-403, doi:10.3390/metabo5020389 (2015).

2 Weir, J. M. *et al.* Plasma lipid profiling in a large population-based cohort. *Journal of lipid research* **54**, 2898-2908, doi:10.1194/jlr.P035808 (2013).

3 Liu, G. *et al.* NetAffx: Affymetrix probesets and annotations. *Nucleic acids research* **31**, 82-86 (2003).

4 Irizarry, R. A. *et al.* Exploration, normalization, and summaries of high density oligonucleotide array probe level data. *Biostatistics* **4**, 249-264, doi:10.1093/biostatistics/4.2.249 (2003).

5 Ritchie, M. E. *et al.* limma powers differential expression analyses for RNA-sequencing and microarray studies. *Nucleic acids research* **43**, e47, doi:10.1093/nar/gkv007 (2015).

6 Ao, S. I. *et al.* CLUSTAG: hierarchical clustering and graph methods for selecting tag SNPs. *Bioinformatics* **21**, 1735-1736, doi:10.1093/bioinformatics/bti201 (2005).

7 Wong, L. P. *et al.* Deep whole-genome sequencing of 100 southeast Asian Malays. *American journal of human genetics* **92**, 52-66, doi:10.1016/j.ajhg.2012.12.005 (2013).

8 Wong, L. P. *et al.* Insights into the genetic structure and diversity of 38 South Asian Indians from deep whole-genome sequencing. *PLoS genetics* **10**, e1004377, doi:10.1371/journal.pgen.1004377 (2014).

9. R Core Team, R: A language and environment for statistical computing. R Foundation for Statistical Computing (2013) Available at: www.R-project.org/, Date of access: 01/08/2015

10. Maechler, M., Rousseeuw, P., Struyf, A., Hubert, M., Hornik, K.(2016). cluster: Cluster Analysis Basics and Extensions. R package version 2.0.5. Date of access: 01/08/2015
